# Predictive worth of estimated glucose disposal rate: evaluation in patients with non-ST-segment elevation acute coronary syndrome and non-diabetic patients after percutaneous coronary intervention

**DOI:** 10.1186/s13098-022-00915-9

**Published:** 2022-10-06

**Authors:** Chi Liu, Xiaoli Liu, Xiaoteng Ma, Yujing Cheng, Yan Sun, Dai Zhang, Qi Zhao, Yujie Zhou

**Affiliations:** 1 Department of Cardiology, Beijing Anzhen Hospital, Beijing Institute of Heart Lung and Blood Vessel Disease, Beijing Key Laboratory of Precision Medicine of Coronary Atherosclerotic Disease, Clinical Center for Coronary Heart Disease, Capital Medical University, Beijing, 100029 China; 2 Department of Cardiology, Cardiovascular Center, Beijing Friendship Hospital, Capital Medical University, Beijing, 100050 China

**Keywords:** Estimated glucose disposal rate, Prognosis, Non-diabetes, Non-ST-segment elevation acute coronary syndrome, Percutaneous coronary intervention

## Abstract

**Background:**

Measurement of estimated glucose disposal rate (eGDR) has been demonstrated to be an indicator of insulin resistance (IR) and a risk sign for long-term outcomes in those with ischemic heart disease and type 2 diabetes mellitus (T2DM) having coronary artery bypass grafting (CABG). After elective percutaneous coronary intervention (PCI), the usefulness of eGDR for prognosis in those with non-ST-segment elevation acute coronary syndrome (NSTE-ACS) and non-diabetes is yet unknown.

**Methods:**

1510 NSTE-ACS patients with non-diabetes who underwent elective PCI in 2015 (Beijing Anzhen Hospital) were included in this study. Major adverse cardio-cerebral events (MACCEs), such as all-cause mortality, non-fatal myocardial infarction, non-fatal ischemic stroke, and also ischemia-driven revascularization, were the main outcome of follow-up. The average number of follow-up months was 41.84.

**Results:**

After multivariate Cox regression tests with confounder adjustment, the occurrence of MACCE in the lower eGDR cluster was considerably higher than in the higher eGDR cluster, demonstrating that eGDR is an independent prognostic indicator of MACCEs. In particular, as continuous variate: hazard ratio (HR) of 1.337, 95% confidence interval (CI) of 1.201–1.488, P < 0.001. eGDR improves the predictive power of usual cardiovascular risk factors for the primary endpoint. Specifically, the results for the area under the receiver operating characteristic (ROC) curve, this is AUC, were: baseline model + eGDR 0.699 vs. baseline model 0.588; P for contrast < 0.001; continuous net reclassification improvement (continuous-NRI) = 0.089, P < 0.001; and integrated discrimination improvement (IDI) = 0.017, P < 0.001.

**Conclusion:**

Low eGDR levels showed a strong correlation with poor NSTE-ACS prognosis for nondiabetic patients undergoing PCI.

**Supplementary Information:**

The online version contains supplementary material available at 10.1186/s13098-022-00915-9.

## Introduction

In recent years, with the widespread application of optimized drug therapy and the improvement of interventional strategies such as revascularization, patients’ prognosis with cardiovascular disease (CVD) has been significantly improved. However, patients with CVD still have a high risk of recurrent cardiovascular events [1–4]. Therefore, identifying remaining risk factors in patients with CVD and expanding new treatment targets are of significant clinical importance. The incidence of CVD and its poor prognosis are strongly predicted by insulin resistance (IR), according to numerous prior investigations [5–7]. It is notable that IR has an equal impact on how CVD develops in those who do not have diabetes [8, 9]. Using the homeostasis model assessment of insulin resistance (HOMA-IR), a meta-analysis of 65 trials with 516,325 individuals revealed that the possibility of coronary heart disease increased by 46% in the non-diabetic for every 1 standard deviation increase in IR [10]. Although the hyperinsulinemic–euglycemic (HIEG) clamp is thought as the most accurate method for detecting IR, clinical researchers are more likely to search for IR assessment methods with broader applicability scenarios due to its complex and invasive characteristics. The estimated glucose disposal rate (eGDR) is an alternative to the HIEG clamp to assess insulin responsiveness when undergoing type 1 diabetes mellitus (T1DM) [11]. Calculation of eGDR is based on waist circumference (WC), hypertension, and glycosylated hemoglobin (HbA1c) [11, 12], which are widely recognized as risk elements for CVD and are readily available from clinical data. The IR level increases when eGDR decreases. Low eGDR has been connected to a higher long-term danger of all-cause death in people with type 2 diabetes mellitus (T2DM) following coronary artery bypass grafting (CABG), suggesting that eGDR may efficaciously indicate poor projection in T2DM patients with ischemic heart disease after revascularization [13]. Considering the economic and efficient characteristics of eGDR, it is suitable to be popularized as a routine screening method for CVD high-risk groups. Thus, we aimed to investigate the connection between eGDR and long-term outlook for CVD nondiabetic patients. We explored the prognostic worth of eGDR in subjects that did not have diabetes but were undergoing non-ST-segment elevation acute coronary syndrome (NSTE-ACS) and had experienced percutaneous coronary intervention (PCI).

## Materials and methods

### Study subjects

**Fig. 1:**
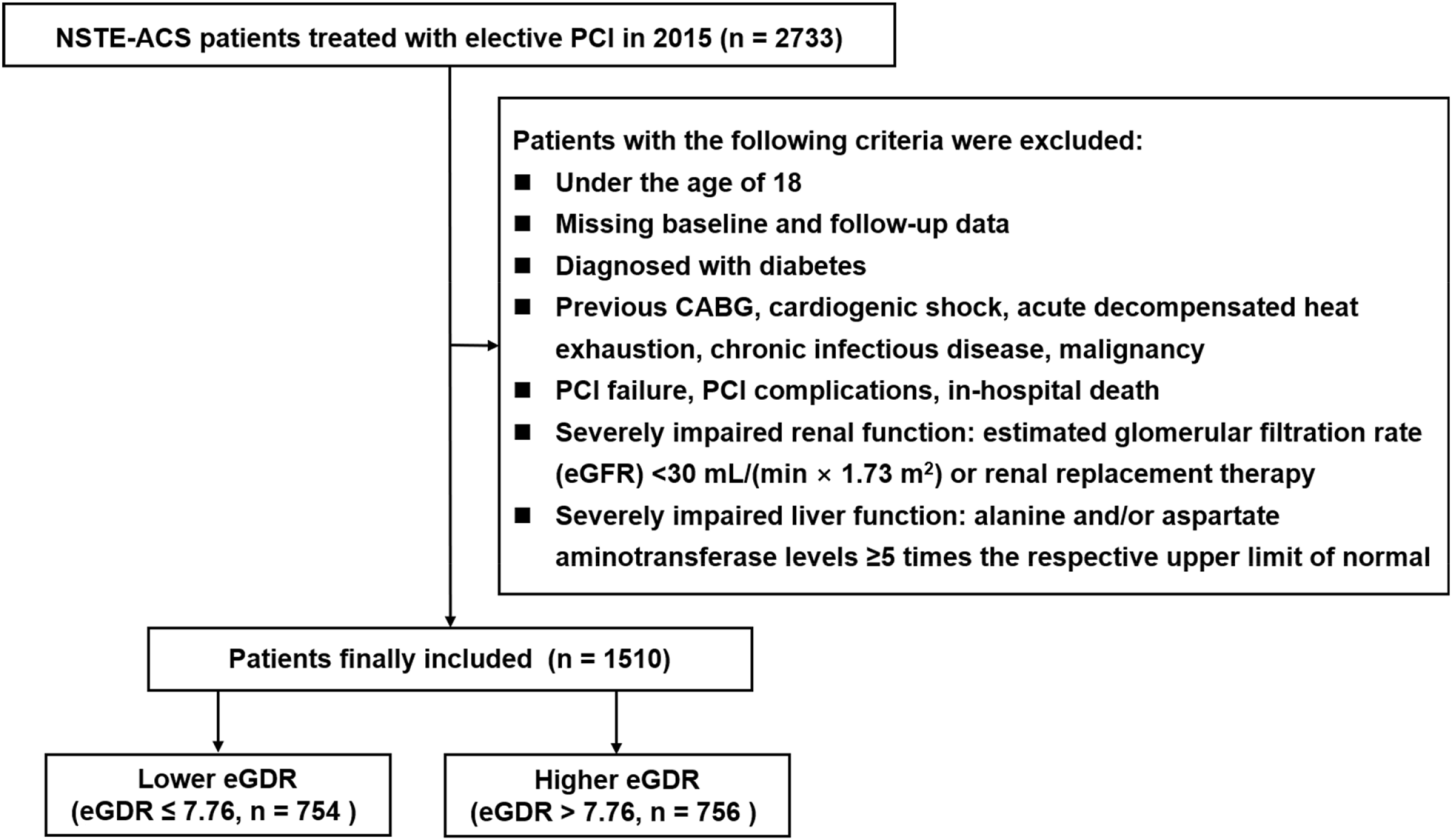
Flow diagram for the enrollment of study population. *NSTE-ACS* Non-ST-segment elevation acute coronary syndrome, *PCI* Percutaneous coronary intervention, *CABG* Coronary artery bypass grafting, *eGFR* estimated glomerular filtration rate, *eGDR* estimated glucose disposal rate

This was a single-center study with an observational cohort. Enrolled subjects had a diagnosis of NSTE-ACS (Beijing Anzhen Hospital, 2015) and underwent elective PCI. Patients with previously or newly diagnosed diabetes at admission were excluded. The diagnostic principles for NSTE-ACS [which involved unstable angina (UA) and non-ST-segment elevation myocardial infarction (NSTEMI)] and diabetes followed the authority’s guidelines [14, 15]. Figure 1 depicts further exclusion requirements. Finally, 1510 patients were enrolled for this investigation.

### Data gathering and descriptions

The baseline facts attained in this study, including demographic data, patient features, laboratory examinations, imaging data, PCI-related data, and medication information, were quality-controlled by the hospital information center. After several measurements taken on various days, hypertension was determined to entail systolic blood pressure (SBP) of over 140 mmHg and/or diastolic blood pressure (DBP) of values below 90 mmHg [16]. Following current recommendations, the diagnostic standards for peripheral arterial disease (PAD), stroke, and dyslipidemia were also employed [17–19]. The WC was the width of the line separating the iliac crest's upper border from the nethermost part of the rib. On the operation day’s morning, blood for hematological and bio-chemical analyses was collected from patients who had fasted for 8–12 h. High-performance liquid chromatography served as a detection method for HbA1c. Two qualified professionals assessed the outcomes of the PCI and echocardiography tests, respectively. Coronary intervention procedures were performed according to the most recent recommendations [20–22]. Using the standard formula found in https://syntaxscore.org, the Synergy between percutaneous coronary intervention with taxus and cardiac surgery (SYNTAX) scores was computed. The severity of coronary lesions was also assessed by the Gensini score [23]. The calculation of eGDR was conducted as [11, 12, 24]: eGDR = 21.16−(0.09*WC [in cm])−(3.41*Hypertension [affirmative or negative])−(0.55*HbA1c [in %]).

### Follow-up and research endpoint

The monitoring period entailed 48 months after hospital release or until the patient died (average monitoring time: 41.84 months). The primary end point was major adverse cardio-cerebral events (MACCEs), counting all-cause mortality, non-fatal myocardial infarction (MI), non-fatal ischemic stroke, and ischemia-driven revascularization. MI shows that the level of creatine kinase or heart troponin is higher than the superior limit in the reference range, and electrocardiogram (ECG) results and/or ischemic factors indicate ischemia in the myocardium. Using magnetic resonance images (MRI) or computed tomography (CT), ischemic lesions that induce nerve injury are what define a stroke. Revascularization of vessels of target and/or non-target nature reveals ischemia-induced revascularization as a result of recurrent or insistent ischemic signs, such as CABG and PCI.

### Statistical evaluation

Included participants were divided into two groups regarding their median eGDR, this is lower eGDR: eGDR ≤ 7.76, and higher eGDR: eGDR > 7.76. The mean standard deviation of continuous variables with normal distribution is displayed and contrasted by a two independent t-test. In the case of the Mann–Whitney U test, it compares continuous variables with skew distributions represented by the median, 25th, and 75th percentiles. Nominal variables were expressed as numbers and percentages, and then contrasted by chi square, continuity-corrected chi square, or using the Fisher's exact.

The Kaplan–Meier curve employed described the growing amounts of the primary endpoint events under diverse eGDR levels, and a log-rank test allowed comparison. Variables that probably had collinearity were eliminated from the 4 multivariate models, which included contained potential risk factors for MACCE that were initially identified in univariate Cox regression analysis. Nominal and continuous variables, respectively, were used to evaluate eGDR. Correlations involved hazard ratios (HR) and 95% confidence intervals (CI). In particular, the multivariate Cox regression models were as follows: in Model 1, adjustments comprised age, sex, body mass index (BMI); Model 2 as Model 1 plus previous MI, previous PCI, previous stroke, smoking history and family history of coronary artery disease (CAD); Model 3 adjusted as Model 2 plus triglyceride (TG), total cholesterol (TC), high-density lipoprotein cholesterol (HDL-C), estimated glomerular filtration rate (eGFR), high-sensitivity C-reactive protein (hs-CRP), angiotensin-converting enzyme inhibitor (ACEI)/angiotensin receptor blocker (ARB) at admission, and left ventricular ejection fraction (LVEF); Model 4 adjusted as Model 3 plus left main artery (LM) lesion, bifurcation, SYNTAX score, multi-vessel lesion, in-stent restenosis, chronic total occlusion lesion, complete revascularization, number of drug-eluting stent (DES), and treatments for: LM, left anterior descending artery (LAD), left circumflex artery (LCX), and right coronary artery (RCA).

In agreement with model 4, a restricted cubic spline curve represents the dose–response connection of eGDR and the primary endpoint. Nonlinear hypotheses are tested using a likelihood ratio analysis. Stratified scrutiny adjusted for model 4 variables considered sex, age, BMI, hyperlipidemia, family history of CAD, smoking history, diagnosis, ACEI/ARB at admittance, and statins at admittance to define the consistency of eGDR in MACCEs prediction. To evaluate eGDR's ability to predict MACCEs, the area under the receiver operating characteristic (ROC) curve, this is, AUC, was obtained. Integrated discrimination improvement (IDI) plus continuous net reclassification improvement (continuous-NRI) confirmed the progressive influence of eGDR introduction on the prognostic competency of presently recognized risk models.

Data examination was performed using SPSS v26.0 and R3.6.3. Statistical significance was acknowledged when two-tailed P < 0.05 was obtained.

## Results

### Patient characterization at the starting point

This study comprised 1510 individuals (mean age = 59.67 ± 9.27) with a 73.7% male participation rate (n = 1113). Age, BMI, WC, SBP, DBP, TG, hs-CRP, HbA1c, FBG, uric acid, creatinine, incidence of hypertension and prior stroke were all higher in the lower eGDR cluster when contrasted to the higher eGDR cluster, whereas HDL-C and eGFR were lower. Patients with lower eGDRs received more ACEI/ARB and β-blocker prescriptions for admission and discharge medication. Participants with low eGDR presented a higher multi-vessel lesions rate and target vessels of LCX and RCA treated, an inferior percentage of complete revascularization, and increased DES implanted. Additionally, the lower eGDR group's SYNTAX score was higher than the higher eGDR group’s (Table 1). A representative case of the extent of coronary artery disease is shown in Additional file 1: Figure S1.

**Table 1:** Baseline characteristics of the study population in two groups of eGDR

	Total population (n = 1510)	Lower eGDR (≤ 7.76; n = 754)	Higher eGDR (> 7.76; n = 756)	*P* value
Age, years	59.67 ± 9.27	60.14 ± 9.20	59.20 ± 9.31	0.048
Sex, male, n (%)	1113 (73.7)	570 (75.6)	543 (71.8)	0.096
BMI, kg/m^2^	25.78 ± 3.15	27.03 ± 2.88	24.53 ± 2.90	< 0.001
WC, cm	89.60 ± 11.96	94.48 ± 10.67	84.75 ± 11.17	< 0.001
Heart rate, bpm	68.62 ± 9.96	68.93 ± 10.18	68.31 ± 9.74	0.233
SBP, mmHg	129.48 ± 16.02	133.03 ± 16.44	125.94 ± 14.79	< 0.001
DBP, mmHg	77.09 ± 9.57	78.70 ± 9.77	75.48 ± 9.10	< 0.001
Smoking history, n (%)	892 (59.1)	460 (61.0)	432 (57.1)	0.127
Drinking history, n (%)	352 (23.3)	179 (23.7)	173 (22.9)	0.694
Family history of CAD, n (%)	143 (9.5)	70 (9.3)	73 (9.7)	0.805
Medical history, n (%)				
Hypertension	863 (57.2)	745 (98.8)	118 (15.6)	< 0.001
Hyperlipidemia	1276 (84.5)	647 (85.8)	629 (83.2)	0.161
Previous MI	309 (20.5)	150 (19.9)	159 (21.0)	0.584
Previous PCI	231 (15.3)	114 (15.1)	117 (15.5)	0.847
Previous stroke	155 (10.3)	102 (13.5)	53 (7.0)	< 0.001
Previous PAD	51 (3.4)	29 (3.8)	22 (2.9)	0.314
Clinical diagnosis, n (%)				0.962
UA	1271 (84.2)	635 (84.2)	636 (84.1)	
NSTEMI	239 (15.8)	119 (15.8)	120 (15.9)	
Laboratory examinations				
TG, mmol/L	1.46 (1.04, 2.03)	1.54 (1.10, 2.11)	1.35 (0.99, 1.93)	< 0.001
TC, mmol/L	4.20 ± 1.03	4.17 ± 1.00	4.24 ± 1.06	0.190
LDL-C, mmol/L	2.55 ± 0.88	2.53 ± 0.86	2.58 ± 0.90	0.351
HDL-C, mmol/L	1.00 ± 0.24	0.98 ± 0.22	1.02 ± 0.25	< 0.001
hs-CRP, mg/L	1.16 (0.52, 2.82)	1.32 (0.61, 3.14)	0.97 (0.45, 2.55)	< 0.001
Creatinine, μmol/L	77.19 ± 16.18	79.52 ± 16.75	74.87 ± 15.25	< 0.001
eGFR, mL/(min × 1.73m^2^)	92.00 ± 18.85	89.43 ± 18.72	94.56 ± 18.64	< 0.001
Uric acid, μmol/L	353.47 ± 82.03	366.25 ± 81.91	340.72 ± 80.19	< 0.001
FBG, mmol/L	5.32 ± 0.60	5.37 ± 0.61	5.27 ± 0.59	0.001
HbA1c, %	5.64 ± 0.39	5.70 ± 0.38	5.59 ± 0.39	< 0.001
LVEF, %	64.03 ± 6.78	64.12 ± 6.47	63.94 ± 7.09	0.590
Medication at admission, n (%)				
ACEI/ARB	304 (20.1)	242 (32.1)	62 (8.2)	< 0.001
DAPT	440 (29.1)	224 (29.7)	216 (28.6)	0.627
Aspirin	793 (52.5)	403 (53.4)	390 (51.6)	0.469
P2Y12 inhibitors	473 (31.3)	238 (31.6)	235 (31.1)	0.841
β-Blocker	339 (22.5)	195 (25.9)	144 (19.0)	0.002
Statins	474 (31.4)	234 (31.0)	240 (31.7)	0.766
Medication at discharge, n (%)				
ACEI/ARB	984 (65.2)	732 (97.1)	252 (33.3)	< 0.001
DAPT	1510 (100.0)	754 (100.0)	756 (100.0)	-
Aspirin	1510 (100.0)	754 (100.0)	756 (100.0)	-
P2Y12 inhibitors	1510 (100.0)	754 (100.0)	756 (100.0)	-
β-Blocker	1351 (89.5)	688 (91.2)	663 (87.7)	0.025
Statins	1469 (97.3)	734 (97.3)	735 (97.2)	0.881
Angiographic data, n (%)				
LM lesion	59 (3.9)	29 (3.8)	30 (4.0)	0.903
Bifurcation	291 (19.3)	140 (18.6)	151 (20.0)	0.489
Multi-vessel lesion	905 (59.9)	498 (66.0)	407 (53.8)	< 0.001
In-stent restenosis	67 (4.4)	33 (4.4)	34 (4.5)	0.909
Chronic total occlusion lesion	182 (12.1)	103 (13.7)	79 (10.4)	0.055
SYNTAX score	9.86 ± 5.25	10.38 ± 5.32	9.34 ± 5.13	< 0.001
Gensini score	30.97 ± 14.39	31.14 ± 14.30	30.79 ± 14.50	0.639
Procedural information				
Target vessel territory, n (%)				
LM	35 (2.3)	15 (2.0)	20 (2.6)	0.397
LAD	993 (65.8)	479 (63.5)	514 (68.0)	0.068
LCX	469 (31.1)	261 (34.6)	208 (27.5)	0.003
RCA	580 (38.4)	313 (41.5)	267 (35.3)	0.013
Complete revascularization, n (%)	949 (62.8)	453 (60.1)	496 (65.6)	0.026
Number of DES	2.00 (1.00, 2.00)	2.00 (1.00, 3.00)	1.00 (1.00, 2.00)	0.002

*eGDR* estimated glucose disposal rate, *BMI* body mass index, *WC* waist circumference, *SBP* systolic blood pressure, *DBP* diastolic blood pressure, *CAD* coronary artery disease, *MI* myocardial infarction, *PCI* percutaneous coronary intervention, *PAD* peripheral artery disease, *UA* unstable angina, *NSTEMI* non-ST-segment elevation myocardial infarction, *TG* triglyceride, *TC* total cholesterol, *LDL-C* low-density lipoprotein cholesterol, *HDL-C* high-density lipoprotein cholesterol, *hs-CRP* HIGH-sensitivity C-reactive protein, *eGFR* estimated glomerular filtration rate, *FBG* fasting blood glucose, *HbA1c* glycosylated hemoglobin A1c, *LVEF* left ventricular ejection fraction, *ACEI* angiotensin-converting enzyme inhibitor, *ARB* angiotensin receptor blocker, *DAPT* dual antiplatelet therapy, *LM* left main artery, *SYNTAX* synergy between PCI with taxus and cardiac surgery, *LAD* left anterior descending artery, *LCX* left circumflex artery, *RCA* right coronary artery, *DES* drug-eluting stent

### Incidence of MACCE

A total of 316 patients (20.9%) experienced MACCE throughout the course of the 48-month follow-up period, including 205 (13.6%) cases of ischemia-induced revascularization, 65 (4.3%) non-fatal myocardial infarctions, 27 (1.8%) non-fatal ischemic strokes, and 19 (1.3%) all-cause mortality. The lower eGDR group had significantly greater incidences of MACCEs (P < 0.001), non-fatal ischemic stroke (P = 0.011), and revascularization due to ischemia (P < 0.001) than the higher eGDR group. Nevertheless, no statistically significant difference was found when the two groups were contrasted in the proportion of all-cause mortality and non-fatal MI (Table 2).

**Table 2:** Incidence of MACCE and each component according to the median of eGDR

	Total population (n = 1510)	Lower eGDR (≤ 7.76; n = 754)	Higher eGDR (> 7.76; n = 756)	*P* value
MACCE, n (%)	316 (20.9)	194 (25.7)	122 (16.1)	< 0.001
All-cause death, n (%)	19 (1.3)	10 (1.3)	9 (1.2)	0.813
Non-fatal MI, n (%)	65 (4.3)	36 (4.8)	29 (3.8)	0.369
Non-fatal ischemic stroke, n (%)	27 (1.8)	20 (2.7)	7 (0.9)	0.011
Ischemia-driven revascularization, n (%)	205 (13.6)	128 (17.0)	77 (10.2)	< 0.001

*eGDR* estimated glucose disposal rate, *MACCE* Major adverse cardio-cerebral events, *MI* Myocardial infarction

### MACCE cumulative risk during follow-up

**Fig. 2:**
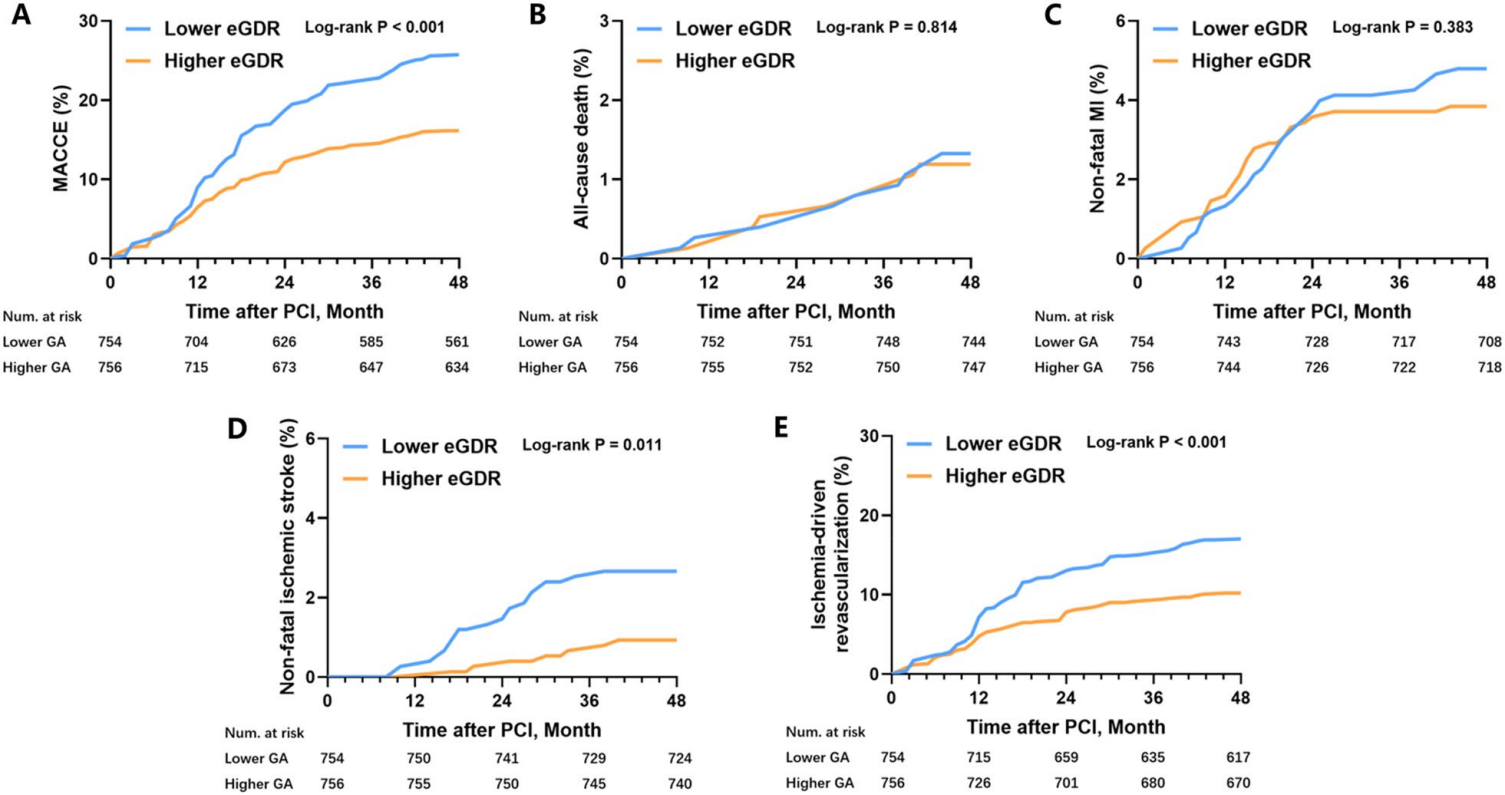
Kaplan–Meier survival curves according to the median of eGDR. **A** Kaplan–Meier survival curve of MACCE; **B** Kaplan–Meier survival curve of all-cause death; **C** Kaplan–Meier survival curve of non-fatal MI; **D** Kaplan–Meier survival curve of non-fatal ischemic stroke; **E** Kaplan–Meier survival curve of ischemia-driven revascularization. *eGDR* estimated glucose disposal rate, *MACCE* major adverse cardio-cerebral events, *MI* myocardial infarction, *PCI* percutaneous coronary intervention

The time-subject cumulative occurrence of MACCE and its components in the two clusters were evaluated using the Kaplan–Meier method. Lower eGDR had a greater cumulative incidence of MACCE (Fig. 2A, log-rank P < 0.001), non-fatal ischemic stroke (Fig. 2D, log-rank was P = 0.011), and ischemia-driven revascularization (Fig. 2E, log-rank was P < 0.001). The cumulative incidence of all-cause mortality (Fig. 2B, log-rank was P = 0.814) and non-fatal MI (Fig. 2C, log-rank was P = 0.383) had no statistical variance between the two clusters.

### Prognostic worth of eGDR for MACCE

To evaluate the eGDR’s ability to predict the primary endpoint, four multivariate models were built (as shown in Methods). Additional file 1: Table S1 summarizes the test of univariate Cox proportional hazards that originally identified predictors of MACCE. After adjusting the variables in the four models, whether eGDR is regarded as a variable either nominal or continuous, it shows substantial independent predictive worth in all models (see Table 3). eGDR was found to be strongly correlated with the possibility of revascularization due to ischemia as a nominal variable and with the possibility of non-fatal MI and revascularization due to ischemia as a continuous variable in the study's further examination of the impact of eGDR in terms of prognosis on each constituent of MACCE (Table 4).

**Table 3:** Predictive value of eGDR for the risk of MACCE

	As nominal variate^a^		As continuous variate^b^	
	HR (95% CI)	*P* value	HR (95% CI)	*P* value
Unadjusted	1.668 (1.330–2.093)	< 0.001	1.194 (1.131–1.260)	< 0.001
Model 1	1.554 (1.213–1.992)	< 0.001	1.260 (1.171–1.357)	< 0.001
Model 2	1.442 (1.125–1.848)	0.004	1.224 (1.137–1.317)	< 0.001
Model 3	1.651 (1.178–2.313)	0.004	1.485 (1.324–1.665)	< 0.001
Model 4	1.557 (1.124–2.158)	0.008	1.337 (1.201–1.488)	< 0.001

Model 1: adjusted for age, sex, BMI

Model 2: adjusted for variates in Model 1 and previous MI, previous PCI, previous stroke, smoking history, family history of CAD

Model 3: adjusted for variates in Model 2 and TG, TC, HDL-C, eGFR, hs-CRP, LVEF, ACEI/ARB at discharge

Model 4: adjusted for variates in Model 3 and LM lesion, bifurcation, multi-vessel lesion, in-stent restenosis, chronic total occlusion lesion, SYNTAX score, LM treatment, LAD treatment, LCX treatment, RCA treatment, complete revascularization, number of DES

*eGDR* estimated glucose disposal rate, *MACCE* major adverse cardio-cerebral events, *HR* hazard ratio, *CI* confidence interval

^a^The HR was evaluated regarding the higher median of eGDR as reference

^b^The HR was evaluated by per 1-unit decrease of eGDR

**Table 4:** Predictive value of eGDR for MACCE and each component in univariate and multivariate analysis

	Univariate analysis			Multivariate analysis^a^		
	HR	95% CI	P value	HR	95% CI	P value
eGDR as a nominal variable^b^						
MACCE	1.688	1.330–2.093	< 0.001	1.557	1.124–2.158	0.008
All-cause death	1.114	0.453–2.742	0.814	0.518	0.135–1.982	0.337
Non-fatal MI	1.246	0.764–2.033	0.377	1.140	0.580–2.241	0.704
Non-fatal ischemic stroke	2.898	1.225–6.853	0.015	0.819	0.260–2.584	0.734
Ischemia-driven revascularization	1.722	1.298–2.285	< 0.001	2.158	1.394–3.342	0.001
eGDR as a continuous variable^c^						
MACCE	1.194	1.131–1.260	< 0.001	1.337	1.201–1.488	< 0.001
All-cause death	1.074	0.871–1.325	0.504	0.776	0.520–1.158	0.214
Non-fatal MI	1.110	0.990–1.245	0.075	1.279	1.027–1.594	0.028
Non-fatal ischemic stroke	1.444	1.173–1.779	0.001	1.791	0.997–3.219	0.051
Ischemia-driven revascularization	1.188	1.112–1.270	< 0.001	1.363	1.190–1.561	< 0.001

*eGDR* estimated glucose disposal rate, *MACCE* major adverse cardio-cerebral events, *HR* hazard ratio, *CI* confidence interval, *MI* myocardial infarction

^a^The multivariate analysis was performed by using Model 4

^b^The HR was evaluated regarding the higher median of eGDR as reference

^c^The HR was evaluated by per 1-unit decrease of eGDR

### Dose–response association of eGDR with MACCE

**Fig. 3:**
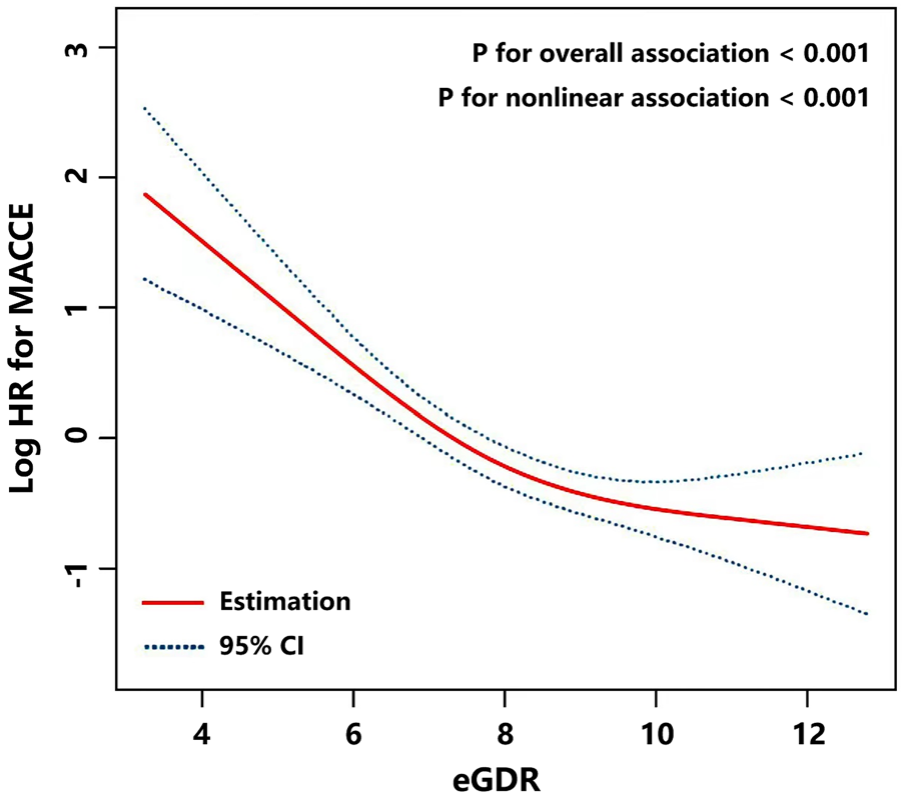
Restricted cubic smoothing for the risk of MACCE according to the eGDR. The analysis was adjusted for Model 4. HR was evaluated by per 1-unit increase of eGDR. *eGDR* estimated glucose disposal rate, *MACCE* major adverse cardio-cerebral events, *CI* confidence interval

A restricted cubic spline curve showed the dose response relationship between eGDR and MACCE (Fig. 3). It was found that the risk of MACCE decreased with the surge of eGDR (P < 0.001). This suggested that eGDR was linearly correlated with the risk of MACCE. A non-linear correlation analysis (P < 0.001) confirmed the above results.

### Stratified analysis of eGDR

**Fig. 4:**
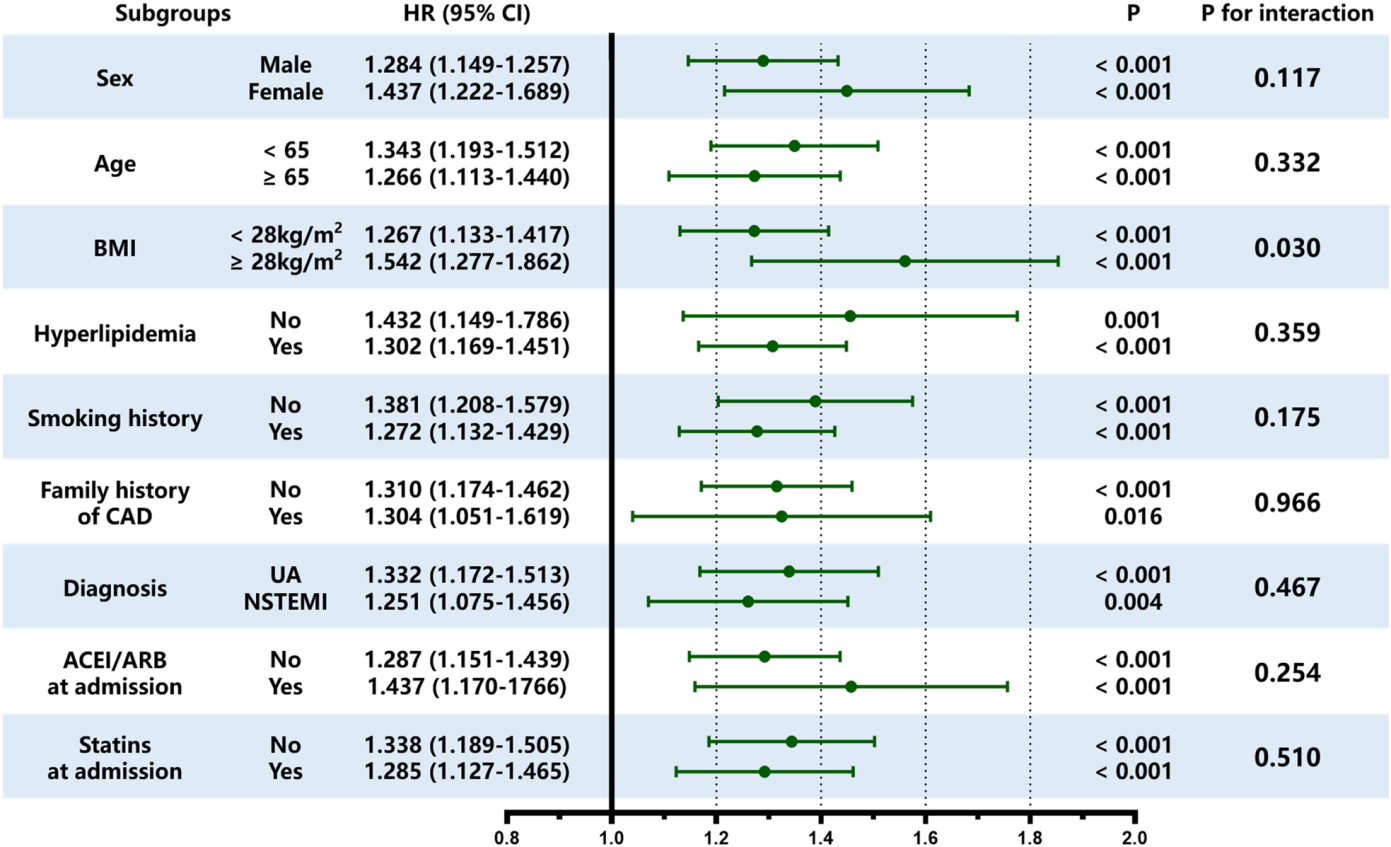
Subgroup analysis evaluating the robustness of eGDR in predicting the risk of the MACCE. The analysis was adjusted for Model 4 except for variates applied for grouping. HR was evaluated by per 1-unit decrease of eGDR. *eGDR* estimated glucose disposal rate, *MACCE* major adverse cardio-cerebral events, *HR* hazard ratio, *CI* confidence interval, *BMI* body mass index, *CAD* coronary artery disease, *UA* unstable angina, *NSTEMI* non-ST-segment elevation myocardial infarction, *ACEI* angiotensin-converting enzyme inhibitor, *ARB* angiotensin receptor blocker

Stratified analysis revealed no difference in the eGDR predictive performance for MACCE considering age (< 65 or ≥ 65 years), sex (male/female), hyperlipidemia (no/yes), smoking history (no/yes), family history of CAD (no/yes), diagnosis (UA or NSTEMI), ACEI/ARB at admittance (on/yes) and statins at admittance (no/yes) (for all, P for interaction > 0.05). More importantly, the eGDR predictive value seemed higher in patients with a higher BMI level [HR (95%CI) BMI < 28 kg/m^2^ 1.267 (1.133–1.417) vs. BMI ≥ 28 kg/m^2^ 1.542 (1.277–1.862), P for interaction = 0.030] (Fig. 4).

### eGDR enhances the prognostic capacities of further parameters in MACCE

**Fig. 5:**
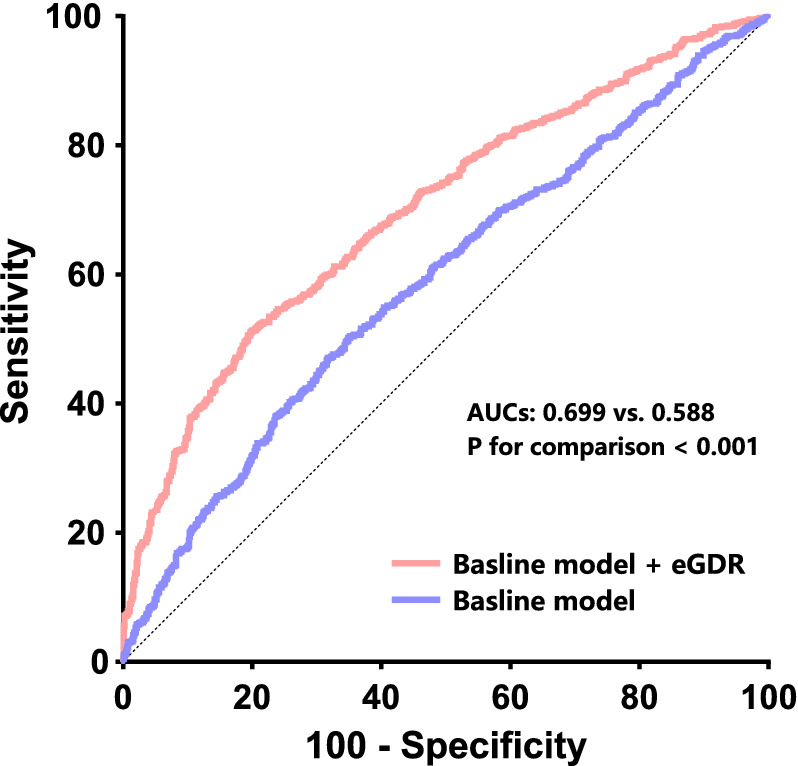
ROC curve to assess the predictive value of eGDR for MACCE. The baseline risk model includes age, sex, BMI, previous MI, previous PCI, previous stroke, hyperlipidemia, smoking history, family history of CAD, eGFR, LVEF, SYNTAX score, complete revascularization. *ROC* receiver-operating characteristic, *eGFR* estimated glomerular filtration rate, *MACCE* major adverse cardio-cerebral events, *AUC* area under curve

On the basis of currently acknowledged cardiovascular risk factors, a baseline model was created (including age, sex, BMI, previous stroke, MI, or PCI, hyperlipidemia, family history of CAD, smoking history, LVEF, SYNTAX score, eGFR, complete revascularization). Adding eGDR significantly enhances the prediction ability of the starting-point model for MACCE (AUCs: baseline model + eGDR 0.699 vs. baseline model 0.588; P for contrast < 0.001) (Table 5; Fig. 5). After adding eGDR, the re-categorizing and discrimination aptitudes considerably outperformed the starting-point risk model (continuous-NRI = 0.089, P < 0.001, IDI = 0.017, P < 0.001) (Table 5).

**Table 5:** Incremental effects of eGDR on risk stratification for MACCE beyond existing risk factors

	ROC curve analysis				Continuous-NRI			IDI		
	AUC	95% CI	P value	*P* for comparison	Estimation	95% CI	*P* value	Estimation	95% CI	*P* value
Baseline model^a^	0.588	0.560–0.617	< 0.001	–	–	–	–	–	–	–
Baseline model + eGDR	0.699	0.672–0.725	< 0.001	< 0.001	0.089	0.037–0.156	< 0.001	0.017	0.007–0.030	< 0.001

*eGDR* estimated glucose disposal rate, *MACCE* Major adverse cardio-cerebral events, *ROC* Receiver-operating characteristic, *NRI* Net reclassification improvement, *IDI* Integrated discrimination improvement, *AUC* Area under curve, *CI* Confidence interval

^a^ Baseline model includes age, sex, BMI, previous MI, previous PCI, previous stroke, hyperlipidemia, smoking history, family history of CAD, eGFR, LVEF, SYNTAX score, complete revascularization

## Discussion

The predictive value of eGDR for unfortunate prognosis in those with NSTE-ACS but no diabetes following PCI is being assessed for the first time in this study. Studies have shown an increment in the incidence frequency of MACCEs in those with low eGDR levels. The decline in eGDR is still a relevant independent forecaster of poor prognosis in the evaluated subjects even after adjusting confounding variables. The ability of baseline models comprising traditional risk factors to forecast the possibility of unfortunate prognosis was greatly enhanced by eGDR.

The development of atherosclerosis in non-diabetic patients was highly correlated with IR evaluated by the gold standard for diagnosing IR, the HIEG clamp [25]. Since HIEG cannot be extensively employed, studies on the relationship between IR and CVD progression and prognosis mostly use HOMA-IR to evaluate IR [8, 9]. HOMA-IR assessment of IR requires the detection of fasting insulin levels in patients. Even diabetic patients who were hospitalized for PCI in the cardiovascular department do not routinely have their fasting insulin levels checked in clinical practice. Moreover, the accuracy of insulin measurement methods is difficult to ensure consistently across laboratories, especially when insulin levels are low. Several investigations have found a slight correlation between HOMA-IR and the level of IR in healthy individuals [26, 27]. As a result, clinical practice is more likely to adopt more operable alternative assessment indicators to assess each patient's level of IR in non-diabetic patients. Studies revealed that IR is frequently characterized by elevated fasting glucose, elevated TG, and obesity in addition to elevated fasting insulin levels (especially increased visceral fat) [28]. Based on these factors, a selection of less complex alternative indicators of IR have been proposed by researchers, such as TG/HDL-C, triglyceride-glucose (TyG) index, visceral adiposity index (VAI), etc., and have been confirmed to be significantly correlated with HIEG clamp [29–31]. Subsequent studies have established that the development and prognosis of diabetes and cardiovascular disease are closely associated to these simple surrogate assessment indicators of IR [32–34]. Studies have indicated that excessive TG/HDL-C levels and the TyG index are independently related to a greater risk of coronary heart disease in non-diabetic patients, while this correlation is not significant in diabetic patients [35]. Compared to the HIEG clamp, eGDR was shown to have similar accuracy. The simplicity of eGDR calculation makes it suitable for large-scale clinical applications. When using as a simple surrogate for assessing IR, eGDR proved a significant correlation with an increased possibility of CVD in T1DM patients [36, 37]. Minor eGDR is associated with an increased possibility of stroke and death in T2DM patients, indicating that eGDR may behave as a predictive marker for these outcomes [38]. Therefore, eGDR is speculated to have good performance in predicting long-standing poor forecast after PCI.

Our study shows that low eGDR is a strong and stable predictor of poor prognosis after PCI in NSTE-ACS and non-diabetic populations. The findings in this study are consistent with previous related studies. Analyzing our findings in terms of pathophysiological mechanisms, as a potent growth factor, the compensatory increase of insulin in the state of IR stimulates the growth, proliferation, and differentiation of vascular smooth muscle cells and activates inflammatory pathways [39]. IR can cause vascular endothelial dysfunction by affecting the activation of nitric oxide, which may be the most important mechanism that causes cardiovascular disease at the cellular level [39, 40]. Therefore, as a simple surrogate index for IR assessment, eGDR can predict the prognosis of patients with cardiovascular disease to a certain extent. On the other hand, eGDR holds three elements: HbA1c, hypertension, and WC. As a recognized traditional risk factor for CVD, hypertension is the most essential constituent of eGDR [11]. In CVD patients with or without diabetes, HbA1c is thought as an independent forecaster of poor outcomes following PCI [41, 42]. Obesity is not only highly correlated with IR [28], but also with maladies such dyslipidemia, CVD, hypertension, and stroke [43]. In patients undergoing PCI, WC is connected with an augmented possibility of cardiac death and non-lethal MI [44]. The release of various cytokines from visceral adipose tissue can lead to inflammation and thrombosis, induce endothelial dysfunction, and accelerate the atherosclerotic process [45, 46]. In the Kaplan–Meier analysis, the differences between the two groups were not statistically significant in all-cause death and non-fatal MI. Because, in this study, patients were predominantly with UA, resulting in a low incidence of adverse events and potentially leading to bias. In addition, it may also be because eGDR is difficult to reflect relatively severe poor prognosis. Notably, in the subgroup analysis, eGDR presented greater predictive worth in the high BMI subcategory (BMI ≥ 28 kg/m^2^) versus the low BMI subgroup (BMI < 28 kg/m^2^). Earlier studies have shown that obesity can cause and exacerbate IR [47]. At the same time, obesity is also a recognized traditional risk factor for CVD. We conjecture that elevated BMI enhances the predictive power of eGDR for long-term outcomes in the study population, but further research is needed to verify this.

There are several limitations to this study as well, which cannot be overlooked. Firstly, it should be considered that this is a single-center, observational study. Therefore, a larger-scale multi-center clinical trial involving more ethnic groups is needed to further validate the conclusions of this study. Secondly, this study did not perform a cross-sectional comparison of eGDR with other simple surrogate metrics for assessing IR. Therefore, future studies need to further clarify the role of eGDR as a predictor of CVD prognosis. Thirdly, since most of the NSTE-ACS patients in this investigation had UA, the predictive value of eGDR in NSTEMI patients may not be accurately reflected by these data. Fourthly, the end points of this study did not include heart failure and cardiac death.

## Conclusions

eGDR proved to be an independent predictor of a poor prognosis in diabetes-negative patients with NSTE-ACS and PCI. The prediction ability that conventional risk variables showed for a poor prognosis was greatly improved by eGDR.

## Supplementary Information

**Additional file 1: Table S1.** Unadjusted Cox regression analysis investigating predictors of MACCE. **Figure S1.** Calculation of Gensini score of representative case.

## Data Availability

The dataset for this study is available from the authors upon reasonable request.

## Abbreviations

CVD: Cardiovascular disease
IR: Insulin resistance
HOMA-IR: Homoeostasis model assessment of insulin resistance
HIEG: Hyperinsulinemic–euglycemic
eGDR: Estimated glucose disposal rate
T1DM: Type 1 diabetes mellitus
WC: Waist circumference
HbA1c: Glycosylated hemoglobin
CABG: Coronary artery bypass grafting
T2DM: Type 2 diabetes mellitus
NSTE-ACS: Non-ST-segment elevation acute coronary syndrome
PCI: Percutaneous coronary intervention
NSTEMI: Non-ST-segment elevation myocardial infarction
UA: Unstable angina
SBP: Systolic blood pressure
DBP: Diastolic blood pressure
PAD: Peripheral arterial disease
SYNTAX: The synergy between PCI with taxus and cardiac surgery
MACCE: Major adverse cardio-cerebral event
MI: Myocardial infarction
ECG: Electrocardiogram
CT: Computed tomography
MRI: Magnetic resonance imaging
HR: Hazard ratio
CI: Confidence interval
BMI: Body mass index
CAD: Coronary artery disease
TG: Triglyceride
TC: Total cholesterol
HDL-C: High-density lipoprotein cholesterol
LVEF: Left ventricular ejection fraction
ACEI: Angiotensin-converting enzyme inhibitor
ARB: Angiotensin receptor blocker
LM: Left main artery
LAD: Left anterior descending artery
LCX: Left circumflex artery
RCA: Right coronary artery
DES: Drug-eluting stent
ROC: Receiver operating characteristic
AUC: Area under curve
Continuous-NRI: Continuous net reclassification improvement
IDI: Integrated discrimination improvement
TyG: Triglyceride-glucose
VAI: Visceral adiposity index
