# Supplementary material for: Predictive worth of estimated glucose disposal rate: evaluation in patients with non-ST-segment elevation acute coronary syndrome and non-diabetic patients after percutaneous coronary intervention
Source: Diabetol Metab Syndr. 2022 Oct 6;14:145. doi: 10.1186/s13098-022-00915-9 (PMC9535978; doi:10.1186/s13098-022-00915-9)
Supplement: Supplementary file 1 — Additional file 1: Table S1. Unadjusted Cox regression analysis investigating predictors of MACCE. Figure S1. Calculation of Gensini score of representative case. [file 13098_2022_915_MOESM1_ESM.docx]

## Table S1. Unadjusted Cox regression analysis investigating predictors of MACCE

|  | MACCE | | |
| --- | --- | --- | --- |
|  | HR | 95% CI | *P* value |
| Age, per 1 years | 1.044 | 1.031-1.051 | < 0.001 |
| Sex, male as reference | 1.197 | 0.940-1.525 | 0.145 |
| BMI, per 1 kg/m^2^ | 1.030 | 0.995-1.066 | 0.094 |
| WC, per 1 cm | 1.037 | 1.028-1.047 | < 0.001 |
| Heart rate, per 1 bpm | 1.001 | 0.990-1.012 | 0.862 |
| SBP, per 1 mmHg | 1.002 | 0.996-1.009 | 0.489 |
| DBP, per 1 mmHg | 0.989 | 0.977-1.000 | 0.057 |
| Smoking history | 1.000 | 0.800-1.252 | 0.998 |
| Drinking history | 0.836 | 0.637-1.099 | 0.199 |
| Family history of CAD | 1.101 | 0.764-1.586 | 0.607 |
| Hypertension | 1.356 | 1.078-1.706 | 0.009 |
| Hyperlipidemia | 1.788 | 1.234-2.590 | 0.002 |
| Previous MI | 2.656 | 2.113-3.338 | < 0.001 |
| Previous PCI | 1.896 | 1.465-2.455 | < 0.001 |
| Previous stroke | 2.402 | 1.819-3.171 | < 0.001 |
| Previous PAD | 1.557 | 0.927-2.616 | 0.094 |
| TG, per 1 mmol/L | 1.347 | 1.204-1.507 | < 0.001 |
| TC, per 1 mmol/L | 1.064 | 0.959-1.181 | 0.240 |
| LDL-C, per 1 mmol/L | 1.040 | 0.920-1.175 | 0.531 |
| HDL-C, per 1 mmol/L | 0.523 | 0.320-0.856 | 0.010 |
| hs-CRP, per 1 mg/L | 1.009 | 0.992-1.026 | 0.313 |
| Creatinine, per 1 μmol/L | 1.008 | 1.002-1.014 | 0.013 |
| eGFR, per 1 mL/(min × 1.73m^2^) | 0.987 | 0.981-0.993 | < 0.001 |
| Uric acid, μmol/L | 1.000 | 0.999-1.001 | 0.930 |
| FBG, per 1 mmol/L | 1.409 | 1.179-1.684 | < 0.001 |
| HbA1c, per 1% | 2.322 | 1.728-3.121 | < 0.001 |
| LVEF, per 1% | 0.955 | 0.942-0.968 | < 0.001 |
| ACEI/ARB at admission | 1.233 | 0.949-1.602 | 0.117 |
| DAPT at admission | 1.375 | 1.091-1.732 | 0.007 |
| Aspirin at admission | 1.237 | 0.990-1.546 | 0.061 |
| P2Y12 inhibitors at admission | 1.398 | 1.114-1.754 | 0.004 |
| Statins at admission | 1.280 | 1.017-1.610 | 0.035 |
| ACEI/ARB at discharge | 1.581 | 1.229-2.033 | < 0.001 |
| Statins at discharge | 1.821 | 0.753-4.406 | 0.184 |
| LM lesion | 2.241 | 1.466-3.426 | < 0.001 |
| Bifurcation | 1.368 | 1.057-1.770 | 0.017 |
| Multi-vessel lesion | 3.502 | 2.623-4.675 | < 0.001 |
| In-stent restenosis | 2.699 | 1.853-3.933 | < 0.001 |
| Chronic total occlusion lesion | 3.226 | 2.507-4.150 | < 0.001 |
| SYNTAX score | 1.143 | 1.123-1.163 | < 0.001 |
| Gensini score | 1.078 | 1.065-1.091 | < 0.001 |
| LM treatment | 2.218 | 1.298-3.791 | 0.004 |
| LAD treatment | 1.074 | 0.849-1.358 | 0.553 |
| LCX treatment | 1.333 | 1.061-1.676 | 0.014 |
| RCA treatment | 1.777 | 1.425-2.216 | < 0.001 |
| Complete revascularization | 0.496 | 0.398-0.619 | < 0.001 |
| Number of DES, per 1 DES | 1.278 | 1.185-1.377 | < 0.001 |

*BMI* body mass index, *WC* waist circumference, *SBP* systolic blood pressure, *DBP* diastolic blood pressure, *CAD* coronary artery disease, *MI* myocardial infarction, *PCI* percutaneous coronary intervention, *PAD* peripheral artery disease, *TG* triglyceride, *TC* total cholesterol, *LDL-C* low-density lipoprotein cholesterol, *HDL-C* high-density lipoprotein cholesterol, *hs-CRP* high-sensitivity C-reactive protein, *eGFR* estimated glomerular filtration rate, *FBG* fasting blood glucose, *HbA1c* glycosylated hemoglobin A1c, *LVEF* left ventricular ejection fraction, *ACEI* angiotensin-converting enzyme inhibitor, *ARB* angiotensin receptor blocker, *DAPT* dual antiplatelet therapy, *LM* left main artery, *SYNTAX* synergy between PCI with taxus and cardiac surgery, *LAD* left anterior descending artery, *LCX* left circumflex artery, *RCA* right coronary artery, *DES* drug-eluting stent

## Figure S1. Calculation of Gensini score of representative case.


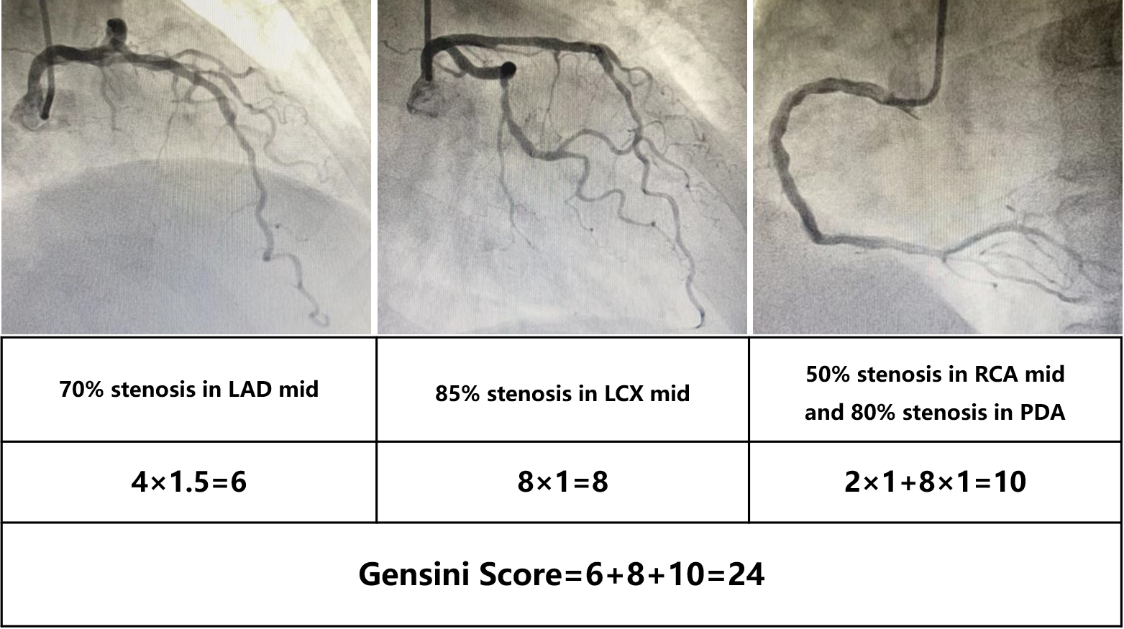


*LAD* left anterior descending artery, *LCX* left circumflex artery, *RCA* right coronary artery, *PDA* [posterior descending artery](http://www.baidu.com/link?url=w4OLzCpHrBD8c1ay-GYacDmCy_duRspVV8ArjSbok5qjmvmxcVaf7kEE81_OKkmPY84xfV22aIEh_OhY7zWd3bH_b-E1_U0QMbAzyAszqTInae3O7lE5dNs1h0_UwtzZxfRm2sUGvhlDuZUdjN6KDK)
